# 16S rDNA sequencing combined with metabolomic probes to investigate the effects of *Salmonella Pullorum* on gut microbes and metabolites in broilers

**DOI:** 10.3389/fmicb.2025.1548782

**Published:** 2025-03-05

**Authors:** Jiongwen Wu, Ruixiang Xue, Zhexia Fan, Ruina Li, Xiaomeng Wang, Chutian Ye, Shuya Chen, Cheng Fang, Xiquan Zhang, Qingbin Luo

**Affiliations:** ^1^College of Animal Science, South China Agricultural University, Guangzhou, China; ^2^State Key Laboratory of Livestock and Poultry Breeding, South China Agricultural University, Guangzhou, China; ^3^Guangdong Provincial Key Lab of Agro-Animal Genomics and Molecular Breeding and Key Lab of Chicken Genetics, Breeding and Reproduction, Ministry of Agriculture, Guangzhou, China

**Keywords:** pullorum disease, *Salmonella pullorum*, cecum, metabolites, microorganisms

## Abstract

Pullorum disease (PD) caused by *Salmonella Pullorum* (SP) results in high mortality in chicks and potential carriers in adult chickens, negatively affecting growth and egg production. This study identified SP infection in 100-day-old White Plymouth Rock hens by serum plate agglutination and fecal and anal swab polymerase chain reaction. SP-infected broilers were classified into positive (P) and negative (N) groups using hematoxylin-and-eosin staining, metabolome sequencing, and 16S rDNA to investigate the effects of SP infection on the metabolites and microorganisms in the cecum of broilers. Groups had different degrees of inflammatory cell infiltration in the cecum, spleen, liver, and lung tissues. The diversity of bacterial flora in the cecum of Groups P and N differed significantly (*P* < 0.05). o__Lactobacillales and o__Verrucomicrobiota were significantly higher in Group P than in Group N (*P* < 0.05). At the genus level, g__*Akkermansia* was significantly higher in Group N (*P* < 0.05). Metabolome sequencing of cecum contents in Groups P and N screened 77 differential metabolites at the secondary metabolite level. 11 metabolites, including 2,4-dimethylbenzaldehyde, 3a,6b,7b,12a-tetrahydroxy-5b-cholanoic acid, and LysoPG 19:1, were differentially expressed in Group P (*P* < 0.05). A combined analysis of 16S rDNA sequencing and cecal content metabolomics identified 28 genera significantly associated with 38 metabolites in the cecum (*P* < 0.05). Specific bacterial genera such as *Corynebacterium* and *Roseobacter* have particularly prominent effects on metabolites. These findings highlight the significant alterations in gut microbial composition and metabolic functions due to SP infection. The differential metabolites and bacterial taxa identified in this study may provide insights into the underlying mechanisms of PD pathogenesis and potential biomarkers for disease management.

## 1 Introduction

Pullorum disease (PD) is an acute systemic disease caused by *Salmonella Pullorum* (SP) infection in the *Salmonella enterica* serotype *enteritidis*. It is spread through various routes, usually horizontally or vertically, in poultry populations, resulting in a significant increase in morbidity and mortality, reduced egg production, and impaired growth, causing significant economic losses to the global farming industry. Especially within 21 days of age, chicks infected with PD exhibit white diarrhea, depression, dehydration, anorexia, and other symptoms, resulting in 100% mortality (Geng et al., 2014). After infection in adult chickens, even if they do not show obvious symptoms, fertility and hatchability are reduced, and SP can colonize the liver, spleen, and intestines for a long time, making adult chickens cryptic carriers (Henderson et al., 1999).

The host's immune system plays a key role in defense against SP infection. However, when the organism cannot completely clear the pathogen, SP can form persistent colonization in the body and spread through the population via fecal-oral transmission or be transmitted to offspring via vertical transmission (Li et al., 2019). Poultry susceptibility and resistance are influenced by genetic background, rearing environment, and immune status (Li et al., 2018).

The SP infection process can be divided into three stages. First, the pathogen invades the intestine via the fecal-oral route and triggers a localized inflammatory response in epithelial cells of the gastrointestinal tract (GIT). Next, SP establishes a systemic infection in gut-associated lymphoid tissues, which involves uptake and transport by macrophages and dendritic cells. After uptake by these immune cells, pathogenic bacteria spread through the lymphatic system to the liver and spleen, triggering a systemic infection. Finally, when the host's infection status is established, the interaction between the SP and host macrophages determines the outcome of the infection: the organism may clear the pathogen through an immune response, or the pathogen may colonize the organ for a prolonged period or even lead to the host's death in severe cases (Chappell et al., 2009).

Usually, SP infections start in the gut, but the poultry gut is a host's important immune organ, with intrinsic immune cells and a rich microbiota working together to maintain gut homeostasis (Kogut and Arsenault, 2016). Changes in the environment, feed, and gastrointestinal microbiota can affect gut health (Shang et al., 2018). Gut health deficits are closely related to microbial imbalance, impaired mucosal barrier, and chronic inflammation (Ducatelle et al., 2018). After SP infection, pathogenic bacteria can spread widely within the GIT, particularly colonizing the ileum and distal region of the cecum, leading to systemic infection. Although the ileum is usually regarded as an important site of immune activation (Simon et al., 2014), *S. enterica* serotype *enteritidis* is more capable of long-term colonization in the cecum and more susceptible to persistent infections than in the ileum (Wigley, 2014).

The intestinal microbial environment is a complex ecosystem known as a “superorganism” that plays a critical role in host immunomodulation, metabolism, and physiological processes (Ranieri et al., 2013). The intestinal microbiota of poultry comprises several microorganism species. The gut microbiota of poultry consists of trillions of microorganisms, predominantly bacteria, with symbiotic functions, which are influenced by the genetic background, site of the digestive tract, diet, and drug use (Kogut, 2022). The microbiota consists of commensal and potentially pathogenic bacteria that play an important role in the animal's health and growth (Choi et al., 2014). Gut homeostasis is dependent on a dynamic balance between the host's immune system and the microbiota, and disruption of this balance can lead to microecological imbalances, such as a decrease in drug-resistant bacteria and an overgrowth of pathogenic bacteria, leading to inflammation and disease (Sun and Jia, 2018). The microbial composition of different intestinal segments varies significantly, but these communities are interconnected and interact with each other to determine the health of the entire GIT (Huang et al., 2018). Therefore, studying the microbiota and metabolic changes in specific intestinal segments, such as the cecum, is important to understand the host's response mechanisms after infection.

Although extensive etiologic and epidemiologic studies on PD have been conducted, the host's molecular mechanisms and metabolic responses during its infection are still understudied. Further excavation of metabolites and molecular markers associated with SP disease resistance may provide an important breakthrough for developing new preventive and therapeutic strategies (Jiang et al., 2023). Studies have shown that SP can colonize the cecum for months and significantly alter local gene expression and microbiota dynamics (Chappell et al., 2009; Barrow, 2000). We employed HE staining, metabolomics analysis, and 16S rDNA sequencing to comprehensively investigate the impact of Salmonella infection on the gut microbiota and metabolites in hens. HE staining provided histopathological evidence to observe inflammation caused by infection; metabolomics analysis revealed metabolic reprogramming induced by the infection and identified differential metabolites associated with intestinal inflammation; and 16S rDNA sequencing illuminated changes in the gut microbiota structure, helping to understand the role of microbial communities in the infection process. The integration of these methods allowed us to explore the infection mechanism from multiple perspectives.

This study aimed to investigate the effects of SP infection on the tissue structure, microbiota, and metabolites of key organs in broilers, including the cecum, spleen, liver, and lungs. The study focused on analyzing the alterations in metabolites and microbial communities within the cecum to elucidate the histopathological characteristics of the host under SP infection and its metabolic adaptation strategies. The findings of this study will provide new insights into the molecular mechanisms of SP infection and establish a theoretical basis for screening resistance markers and developing preventive and control strategies for PD.

## 2 Materials and methods

### 2.1 Identification of test animals and chickens with SP

The hens were provided by Guangzhou Jiangfeng Seed Industry Technology Co., and all of them were selected from the same flock. Additionally, all hens were raised under identical environmental conditions, including standardized feed, temperature, humidity, and light cycles. Blood was collected from 2,100 100-day-old White Plymouth Rock hens using a single-use sterile disposable syringe (1 mL), with 0.5–0.8 mL of blood drawn per hen. The syringe was placed with the needle facing up in an ice box for 1 h to allow serum separation, and the separated serum was then transferred into a 1.5 mL EP tube. The serum was subsequently tested for SP infection using the rapid plate agglutination (RPA) test. Samples with agglutination exceeding 50% were classified as strongly positive. Samples with agglutination but < 50% were classified as weakly positive, and those without agglutination were classified as negative. According to the RPA results, six strongly positive (P) and six negative (N) individuals were randomly selected and housed in single cages, and a fresh anal swab sample and 1–2 g of fresh fecal samples were collected, followed by polymerase chain reaction (PCR) identification. Chickens with identical plate agglutination and PCR results were divided into Groups P and N.

In RPA experiments, serum samples were collected for SP detection. Blood was allowed to stand and coagulate, and the serum was separated. Subsequently, serum and antigen were added at a 1:1 ratio to a 96-well plate. After mixing, the plate was shaken for 2 to 5 min at room temperature and observed for granular agglutination. If there was obvious granular agglutination in the serum but no change in the control saline, it was considered positive, indicating the presence of specific antibodies against SP in the serum; if there was no obvious agglutination in both, it was considered negative. All antisera were stored at 2 to 8°C, avoiding repeated freezing and thawing to ensure activity.

Fecal DNA was extracted according to the instructions of the Fecal DNA Extraction Kit provided by Kangwei Reagent. The extracted DNA was then used as a template for subsequent PCR, with the PCR system and reaction conditions outlined in Table 1. PCR amplification was performed using a T100 PCR machine (Bio-Rad, USA).

**Table 1:** PCR system for SEEP400405.

**Reagents**	**Additive quantity**	**Reaction condition**
SEEP400405-F	0.5 μL	Preheat at 95°C for 5 min
SEEP400405-R	0.5 μL	Denaturation at 95°C for 30 s
2 × Taq PCR MasterMix	10 μL	55°C return 30 s
Ultrapure water	8 μL	72°C extension for 30 s
Template DNA	1 μL	Final extension at 72°C for 5 min
Total	20 μL	4°C storage

For DNA extraction of anal swab samples, 1 mL SC enrichment solution containing the anal swab samples was transferred to a 1.5 mL sterile centrifuge tube and centrifuged at 10,000 rpm for 3 min at room temperature (25°C), and the supernatant was discarded. Then, 1 mL sterile water was added and centrifuged at 10,000 rpm for 3 min at room temperature, and the supernatant was discarded. Next, after resuspension with 50 μL sterile water, the centrifuge tubes were heated in a water bath preheated to 100°C for 10 min. Immediately after heating, the tubes were cooled in an ice box. The cooled samples were centrifuged again at 10,000 rpm for 10 min, and the supernatant was used as the template for PCR.PCR system and reaction conditions are shown in Table 2.

**Table 2:** PCR system for STN and SEP.

**Reagents**	**Additive quantity**	**Reaction condition**
Primers SEP-F and SEP-R	0.5 μL each	Preheat at 95°C for 10 min
Primers STN-F and STN-R	0.5 μL each	Denaturation at 94°C for 30 s
2 × Taq PCR MasterMix	10 μL	57°C return 30 s
Ultrapure water	6 μL	72°C extension for 30 s
Template DNA	2 μL	Final extension at 72°C for 10 min
Total	20 μL	4°C storage

PCR primers were designed with reference to the Lu et al. (2021) study and the Sichuan local standard SP purification counting specification for breeder farms (DB51/T 2665-2019). The primers were synthesized by Beijing Prime Biotechnology Co. Specific primer information is shown in Table 3.

**Table 3:** Specific for SP in chickens.

**Primer name**	**Primer sequence**	**Product length (bp)**
SEEP400405-F	GAGAATCCGGGACGGATGAC	576
SEEP400405-R	CACTCGACAGGAACGCATTG	
STN-F	CTTTGGTCGTAAAATAAGGCG	260
STN-R	TGCCCAAAGCAGAGAGAGATTC	
SEP-F	CACTGGAGACTCTGAGGACAA	537
SEP-R	GGATTGATGAGGCTAACAAGGA	

### 2.2 Tissue sample collection and processing

On the sampling day, 12 hens (6 with confirmed strong positive results and 6 with negative results) were randomly selected for sampling. Tissues from the cecum, spleen, liver, and lungs were collected and fixed in 4% paraformaldehyde for subsequent histological examination. The remaining cecal tissue samples and contents were preserved in crystals and temporarily frozen in liquid nitrogen for further analysis. The metabolomics analysis and 16S rDNA sequencing were conducted by LC-Bio Technologies (Hangzhou) Co., Ltd. The preparation of tissue sections and hematoxylin-and-eosin (H&E) staining were carried out by Wuhan Pinuofei Biotech Co., Ltd.

### 2.3 16S rDNA from the cecum contents

Total microbial DNA was extracted from cecal content samples using the CTAB method. The quality of DNA extraction was assessed by agarose gel electrophoresis, and DNA quantification was performed via ultraviolet spectrophotometry. After PCR amplification of the target fragments, the amplicons were detected by 2% agarose gel electrophoresis and purified. For sequencing, an eligible library was constructed by gradient dilution and mixed according to the required sequencing volume ratio. Subsequently, double-stranded DNA was denatured with NaOH to produce single strands, which were then sequenced.

After sequencing, raw data were obtained, and the overlap between paired-end reads was used to assemble the data. Quality control (QC) and chimera filtering were performed to generate high-quality CleanData.DADA2 v1.24.0 (Divisive Amplicon Denoising Algorithm) obtains representative sequences with single-base accuracy through steps such as dereplication (equivalent to 100% similarity clustering) (Callahan et al., 2016). The DADA2 core was denoised, and the operational taxonomic unit class was constructed using the AMP concept (Blaxter et al., 2005) to obtain the final amplicon sequence variant (ASV) feature table and feature sequences for further diversity analysis, species taxonomic annotation, and differential analysis. 16S amplified fragments and V3–V4 PCR system are shown in Tables 4, 5.

**Table 4:** 16S amplified fragments.

**Amplified fragments**	**Primer name**	**Primer sequence 5^′^-3^′^**
V3-V4	341F	CCTACGGGNGGCWGCAG
	805R	GACTACHVGGGTATCTAATCC

**Table 5:** V3–V4 PCR system.

**Reagents**	**Dosage**
Phusion Hot start flex 2 × Master Mix	12.5 μL
Forward primer, reverse primer	2.5 μL
Template DNA	50 ng
Double-distilled H_2_O	Up to 25 μL

### 2.4 Untargeted metabolomics analysis of cecal contents

Metabolite extraction from the samples (6 in P group and 6 in N group) was conducted using protein precipitation with organic reagents, and QC samples were prepared by pooling the preparative experimental samples. The extracted samples were randomized for mass spectrometry (MS) analysis, with QC samples inserted at the beginning, middle, and end of the run to assess technical reproducibility. MS was performed in the positive and negative ion modes.

Raw MS data were converted to a readable mzXML format using the MSConvert software from Proteowizard. Peak extraction and QC were conducted with XCMS software, followed by ion annotation using CAMERA for the extracted compounds. Metabolite identification was achieved using the metaX software, with primary MS data matched to public databases and secondary MS data cross-referenced with an in-house standard library. Annotated candidate metabolites were further identified in HMDB, Kyoto Encyclopedia of Genes and Genomes (KEGG), and other databases to elucidate their physicochemical properties and biological functions. Differential metabolites were quantified and screened using the metaX software for further analysis.

### 2.5 Integrative analysis of 16S rDNA and untargeted metabolomics

The Spearman correlation coefficient is a statistical measure that evaluates the strength of the monotonic relationship between two variables. In this study, Spearman correlation analysis was applied to assess the associations between differential secondary metabolites identified in the metabolomics analysis and differential genera identified in the 16S rDNA analysis. Based on the results, appropriate filtering criteria were set to construct correlation networks and visualize the relationships between these differential metabolites and microbial taxa.

## 3 Results

### 3.1 *Salmonella pullorum* detection

**Figure 1:**
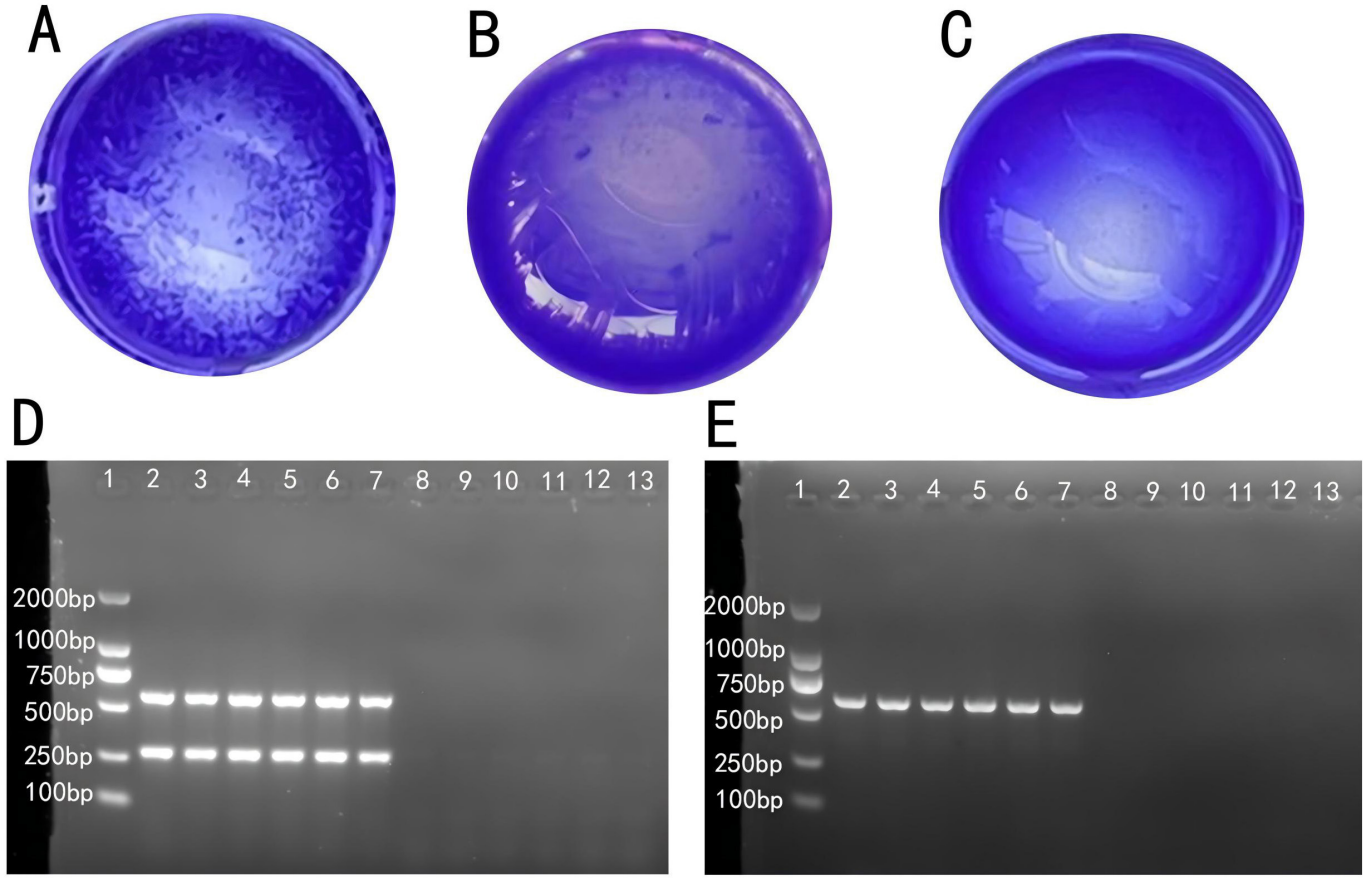
Determination of SP infection in laying hens: **(A)** strong positive, **(B)** weak positive, and **(C)** negative. **(D)** Electrophoresis of the PCR product from anal swab samples. **(E)** Electrophoresis of the PCR product from fecal samples.

The RPA test results determined individuals as strongly positive (Figure 1A), weakly positive (Figure 1B), or negative (Figure 1C). The results showed that 217 out of 2,100 hens were positive, with a positive rate of 10.3%. Samples with ambiguous results were excluded from further analysis.

PCR amplification products from fecal and cloacal swab samples were observed via gel electrophoresis and imaged with a gel imaging system. Figure 1D shows that individuals in lanes 2 to 7 were initially identified as Salmonella-positive, while those in lanes 8 to 13 were negative. Figure 1E further confirmed that individuals in lanes 2 to 7 were infected with S. Pullorum, assigning them to the P group, while those in lanes 8 to 13 were assigned to the N group. Individuals with consistent results across plate agglutination, fecal sample, and cloacal swab PCR product electrophoresis were classified into Groups P and N, which were used for subsequent sequencing.

### 3.2 H&E staining

H&E staining was performed on the cecum, spleen, liver, and lung tissues from individuals in Groups P and N and observed at × 20 magnification. PD had significant pathological effects on broiler tissues, manifesting as epithelial shedding and inflammatory cell infiltration in the intestinal tissue, indicative of intestinal damage and inflammatory response. The spleen tissue displayed a disrupted follicular structure, indistinct boundaries between the red and white pulp, and hemorrhage, reflecting impaired immune function. In the liver, mild hemorrhage and inflammatory cell infiltration indicated slight hepatic damage. Lung tissue exhibited fat vacuoles and perivascular inflammatory cell infiltration accompanied by extensive hemorrhage. These findings collectively demonstrated that PD induces structural abnormalities and dysfunction across multiple organs, severely compromising the overall health of the chickens.

**Figure 2:**
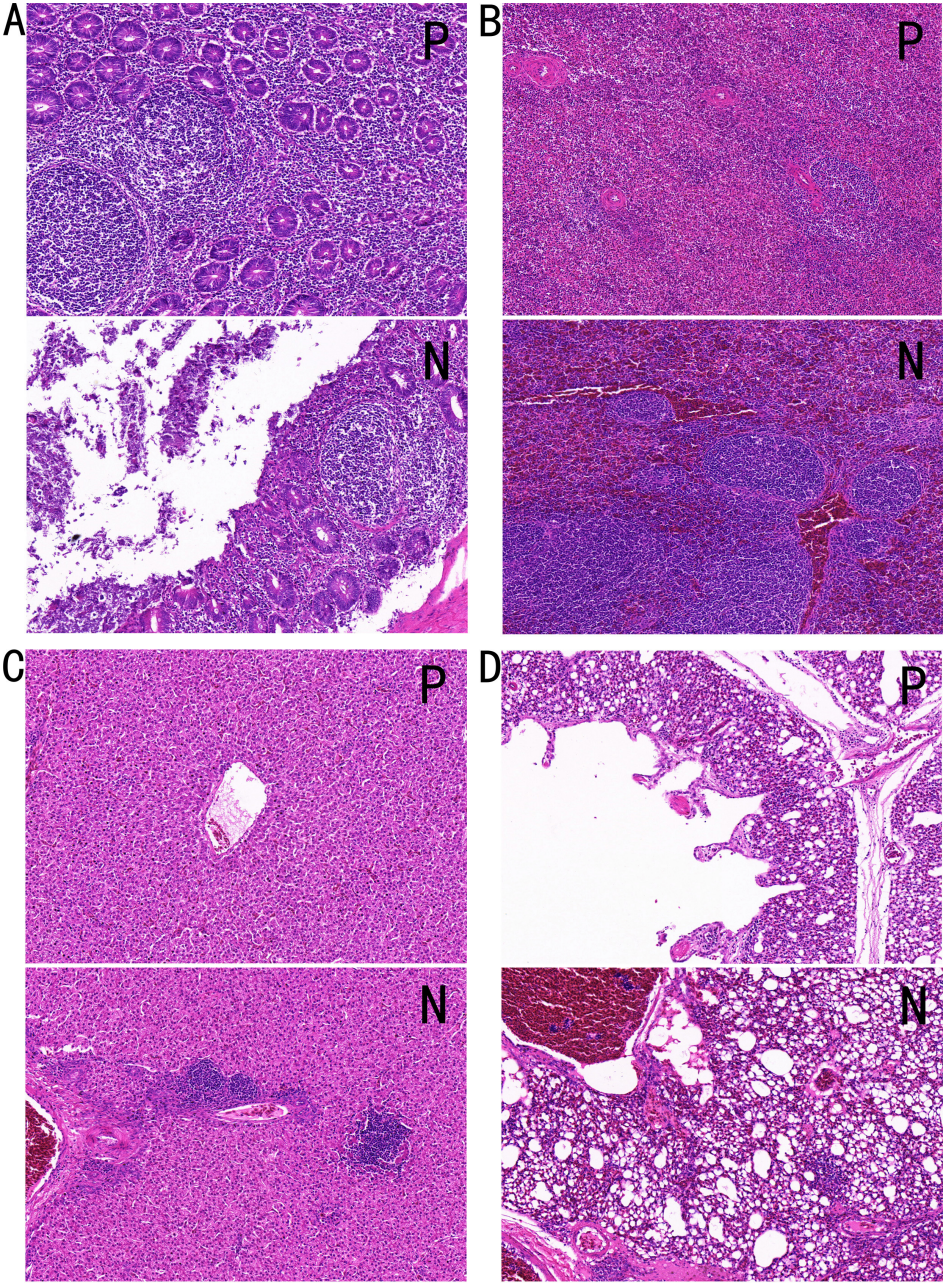
Tissue HandE staining sections. **(A)** Cecal tissue, **(B)** spleen tissue, **(C)** liver tissue, and **(D)** lung tissue. P, Group P; N, Group N.

In Figure 2A, the intestinal tissue structure of negative individuals was essentially normal, with no mucosal epithelial shedding, evenly distributed intestinal glands, and a stable number of crypts, as indicated by red arrows. Lymphoid nodules appear non-enlarged, as denoted by blue arrows. In contrast, the intestinal tissue of positive individuals exhibited moderate structural abnormalities, with disordered mucosal epithelial alignment and significant epithelial erosion and shedding, as shown by yellow arrows. The mucosal layer is heavily infiltrated by inflammatory cells, as indicated by black arrows, and lymph nodes within the mucosal layer are marked by blue arrows.

Figure 2B shows that the spleen tissue structure of negative individuals is generally normal, with an even distribution of red and white pulp, no apparent atrophy, and no reduction in lymphocyte count. Black arrows indicate the white pulp, red arrows indicate the red pulp, and yellow arrows point to the central artery of lymphoid follicles. In contrast, the spleen tissue of positive individuals showed moderate abnormalities, with disordered splenic nodule structures, indistinct boundaries between the red and white pulp, and slight increases in neutrophils in the red pulp, as marked by black arrows. Extensive hemorrhage within the splenic parenchyma is indicated by red arrows.

In Figure 2C, the liver tissue structure of negative individuals appears normal, with well-organized hepatic plates, normal hepatocyte morphology, and uniform cytoplasmic staining, and no obvious hemorrhage is observed. The liver tissue of positive individuals showed mild structural abnormalities, including underdeveloped interlobular connective tissue, hepatic plates formed by two layers of hepatocytes, and well-organized hepatic plates. Mild hemorrhage is noted in blood vessels, with a small number of red blood cells in the tissue, as shown by red arrows. Inflammatory cell infiltration was observed in hepatocyte spaces and around blood vessels, as indicated by black arrows.

Figure 2D shows that the lung tissue structure of negative individuals is generally normal, with clearly defined alveolar structures and no signs of collapse or atrophy, as indicated by yellow arrows. Mild hemorrhage is noted within tissue blood vessels, as shown by red arrows, and no significant inflammatory cell infiltration is observed. Blue arrows denote the smooth muscle. In positive individuals, the lung tissue exhibited mild abnormalities, with scattered fat vacuoles within the tissue, as indicated by yellow arrows. Inflammatory cell infiltration was observed around blood vessels and bronchi, as shown by black arrows. Extensive hemorrhage is visible within tissue spaces, blood vessels, and secondary bronchi, as indicated by red arrows.

### 3.3 Analysis of the microbial community composition of cecal contents

#### 3.3.1 ASV characteristics and α- and β-diversity analysis results

**Figure 3:**
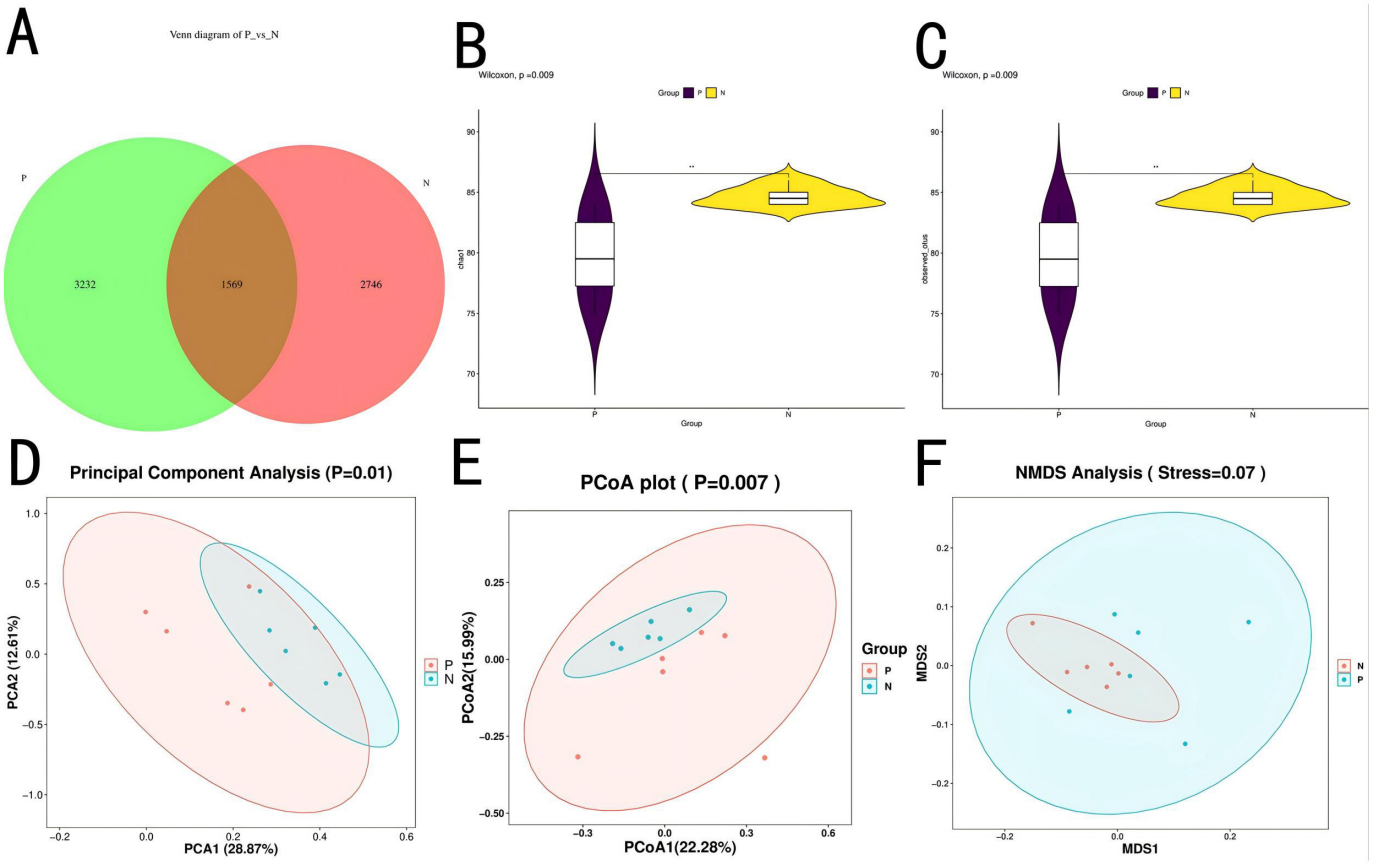
ASV and diversity analysis. **(A)** Venn diagram of ASVs. **(B)** Chao1 index plot. **(C)** Observed species index plot. **(D)** PCA plot. **(E)** PCoA plot. **(F)** NMDS plot.

To investigate the impact of PD on the gut microbiota, 16S rDNA sequencing was performed on cecal contents (*n* = 12). Results revealed 3232 ASVs unique to Group P and 2746 ASVs unique to Group N, with 1569 ASVs shared between both groups, as shown in Figure 3A. Alpha diversity analysis showed that Chao1 and Observed-species index were significantly different between P group and N group (*p* < 0.01), as shown in Figures 3B, C.

α-Diversity analysis indicated a highly significant difference (*P* < 0.01) in the Chao1 and observed species indices between Groups P and N, suggesting that microbial species richness was lower in Group P. β-Diversity analysis, as shown in Figures 3D–F, demonstrated significant separation between groups (*P* < 0.05) across three methods—principal component analysis (PCA), principal coordinate analysis (PCoA), and non-metric multidimensional scaling (NMDS), indicating low similarity and high differentiation in microbial composition between the two groups. NMDS analysis yielded a stress value of 0.07, suggesting a well-fitted model, further supporting significant differences in the microbial community structure between the groups. These variations reflect the disruption of the gut microbiota by PD, resulting in a redistribution of the community structure.

#### 3.3.2 Species composition and gut microbiota functional analysis

**Figure 4:**
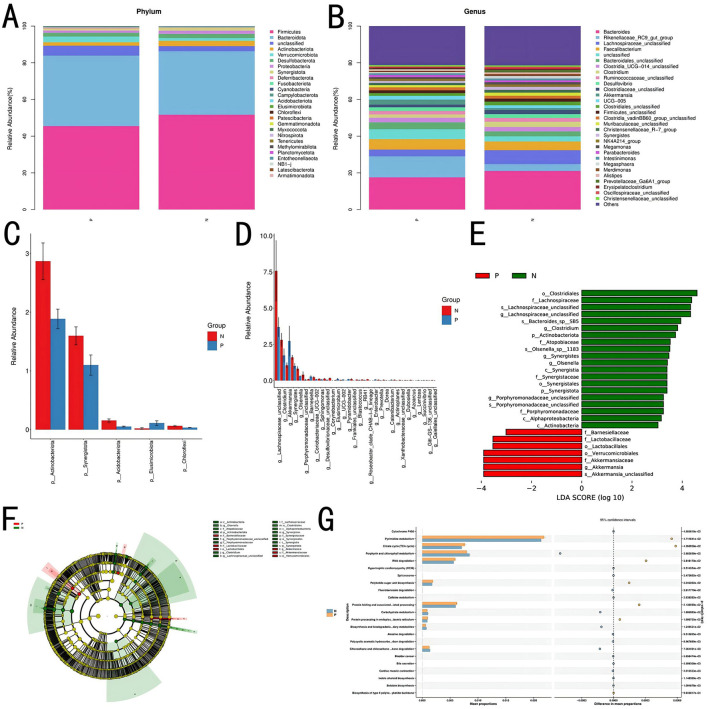
Species composition and gut microbiota functional analysis. **(A)** Correlation analysis at the phylum level. **(B)** Correlation analysis at the genus level. **(C)** Significance analysis of intergroup microbial differences at the phylum level. **(D)** Significance analysis of intergroup microbial differences at the genus level. **(E, F)** Differential analysis using LEfSe. **(G)** Functional prediction and STAMP differential analysis.

Species composition analysis between Groups P and N identified 26 bacterial phyla at the phylum level. Table 6 shows the bacterial phyla with a relative abundance of 1% in the P and N groups, and Table 7 shows the bacterial genera with the highest relative abundance in the P and N groups (top 10). As shown in the Figures 4A–D, the relative abundance of Bacteroidota and Verrucomicrobiota was higher in Group P than in Group N, whereas Firmicutes, Actinobacteria, Desulfobacterota, Proteobacteria, and Synergistota had higher relative abundances in Group N than in Group P. At the genus level, 536 bacterial genera were identified. Genera such as Rikenellaceae_RC9_gut_group, *Faecalibacterium, Clostridia*_UCG-014_unclassified, and *Akkermansia* showed higher relative abundances in Group P, whereas *Bacteroides*, Lachnospiraceae_unclassified, *Clostridium*, Ruminococcaceae_unclassified, *Desulfovibrio*, and Clostridiaceae_unclassified were more abundant in Group N.

**Table 6:** Phyla with 1% relative abundance in groups P and N.

**Fungi phylum**	**Group P (%)**	**Group N (%)**
Firmicutes	45.50	51.69
Bacteroidota	38.37	34.57
Actinobacteriota	1.89	2.87
Verrucomicrobiota	3.06	1.56
Desulfobacterota	2.08	2.24
Proteobacteria	1.25	1.73
Synergistota	1.10	1.60

**Table 7:** Relative abundance of the top 10 genera in Groups P and N.

**Genus *Mycoplasma***	**Group P (%)**	**Group N (%)**
*Bacteroides*	17.56	21.06
Rikenellaceae_RC9_gut_group	11.45	3.72
Lachnospiraceae_unclassified	3.69	7.57
*Faecalibacterium*	5.65	4.74
Clostridia_UCG-014_unclassified	2.53	2.40
*Clostridium*	1.73	2.81
Ruminococcaceae_unclassified	2.00	2.17
*Desulfovibrio*	1.93	2.02
Clostridiaceae_unclassified	1.53	2.27
*Akkermansia*	2.73	1.05

Linear discriminant analysis (LEfSe) indicated that bacterial groups enriched in Group N, including Clostridiales, Lachnospiraceae, and Synergistaceae, likely contributed to maintaining gut microbial stability. In contrast, Group P was enriched with taxa such as Barnesiellaceae, Lactobacillales, and *Akkermansia*, which may be associated with altered gut conditions or disease states, as shown in Figures 4E, F.

Using PICRUSt2 for functional prediction and BugBase for bacterial phenotype prediction, a correlation between the cecal gut microbiota and microbial functions was established. Results suggested potential associations with various functions, including the biosynthesis of type II polyketide backbones, betalain biosynthesis, bile secretion, degradation of chloroalkane and chloroalkene, degradation of polycyclic aromatic hydrocarbons, biosynthesis and biodegradation of secondary metabolites, carbohydrate metabolism, the citrate cycle (tricarboxylic acid cycle), polyketide sugar unit biosynthesis, protein folding and associated processing, protein processing in the endoplasmic reticulum, pyrimidine metabolism, and RNA degradation.

Figure 4G presents the top 30 functions, with significant differences (*P* < 0.05) between Groups P and N, as identified through pairwise *t*-tests.

### 3.4 Comparative analysis of differential metabolites in the gut of PD-positive and -negative chickens

**Figure 5:**
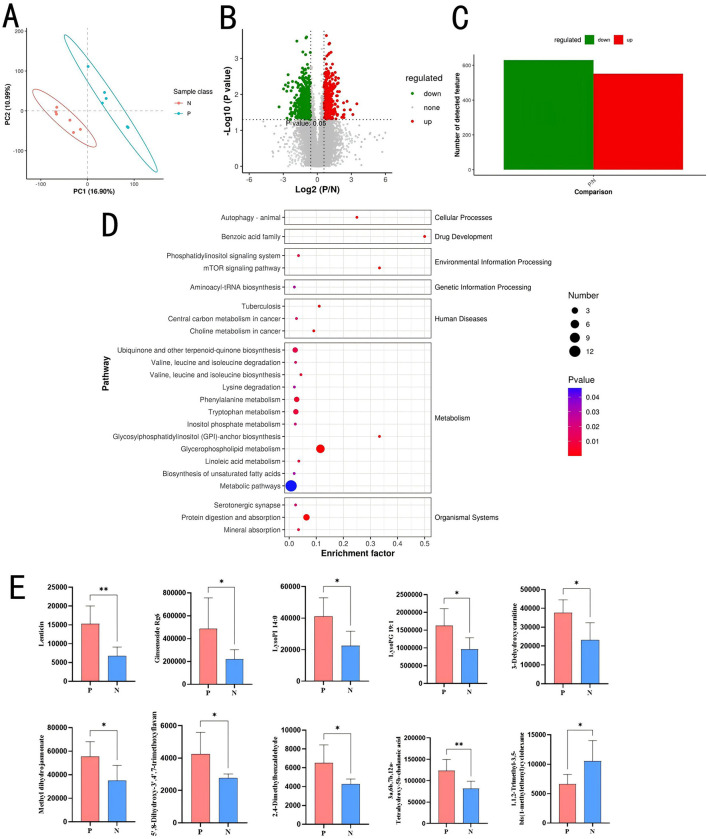
Metabolomic analysis of cecal contents. **(A)** Partial least-squares discriminant analysis plot. **(B)** Volcano plot of differential metabolites. **(C)** Bar chart of differential metabolites. **(D)** KEGG bubble plot of differential metabolites. **(E)** Top 10 differential metabolites. *P < 0.05; **P < 0.01.

To investigate the effects of PD on gut metabolites, metabolomic sequencing was performed on cecal contents of Groups P and N (*n* = 12). The PLS-DA plot (Figure 5A) shows clear separation between the two groups along PC1 (16.90%) and PC2 (10.99%), indicating significant differences in their metabolic or microbial profiles. Over 20,000 compounds were identified, with 516 metabolites upregulated and 665 metabolites downregulated in Group P compared to Group N (Figures 5B, C). At the secondary metabolite level, 77 differential metabolites were identified, with 11 metabolites upregulated in Group P and 66 upregulated in Group N. Metabolites such as 2,4-dimethylbenzaldehyde, 3a,6b,7b,12a-tetrahydroxy-5b-cholanoic acid, LysoPG 19:1, LysoPI 14:0, ginsenoside Rg6,3-dehydroxycarnitine, methyl dihydrojasmonate, lenticin, deoxyadenosine monophosphate, 5′,8-dihydroxy-3′,4′,7-trimethoxyflavan, and docosatrienoic acid were highly expressed in Group P. Figure 5E shows the top 10 metabolites with differences.

KEGG enrichment analysis (Figure 5D) of differential metabolites revealed significant enrichment in pathways such as Glycerophospholipid metabolism (map00564; *P* = 0.0000), Protein digestion and absorption (map04974; *P* = 0.0001), Tryptophan metabolism (map00380; *P* = 0.0080), Phenylalanine metabolism (map00360; *P* = 0.0057), and Ubiquinone and another terpenoid-quinone biosynthesis (map00130; *P* = 0.0107).

### 3.5 Correlation analysis of 16S rDNA and metabolomics

**Figure 6:**
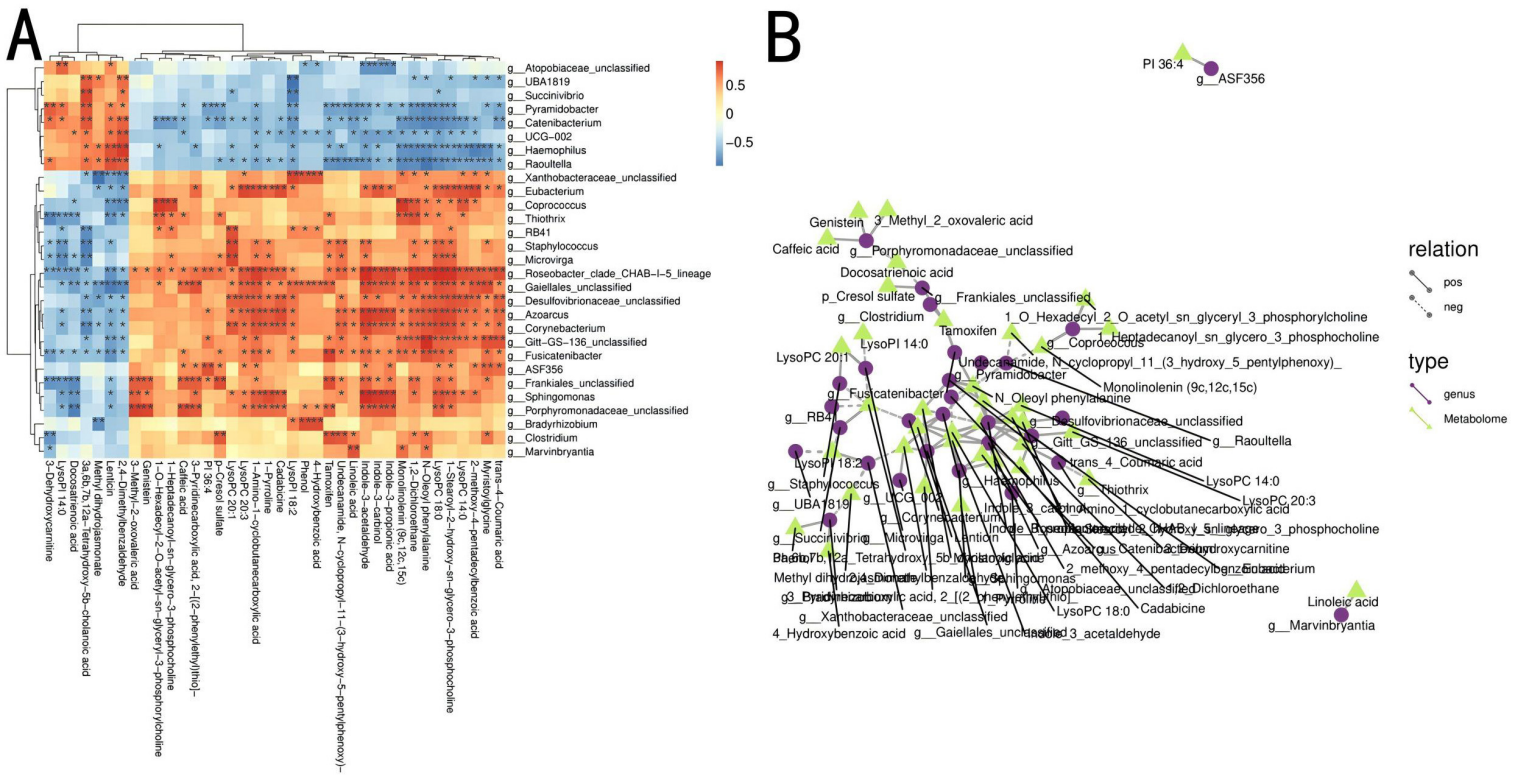
Combined analysis of differential metabolites and microbial communities between Groups P and N. **(A)** Heatmap of correlations between metabolites and differential microbial communities. *P < 0.05; **P < 0.01. **(B)** Network diagram of metabolites and differential microbial communities. Different nodes represent distinct microbial communities and metabolites, with circles representing gut microbiota and triangles representing gut metabolites. Solid lines indicate positive correlations between the gut microbiota and metabolites, whereas dashed lines indicate negative correlations.

To investigate the relationship between the gut microbial communities and the host's metabolite phenotypes and understand how microbial communities influence metabolic functions, a combined analysis of 16S rDNA and cecal content metabolomics was conducted. Spearman correlation coefficients were calculated between 47 differential genera and 68 potential metabolites at the genus level. The results are shown in Figures 6A, B. 28 genera were significantly correlated with 38 cecal metabolites (*P* < 0.05).

*Corynebacterium* positively correlated with phosphocholine and indole-3-acetaldehyde and negatively correlated with tetrahydroxycholanic acid. *Roseobacter* was positively correlated with indole-3-propionic acid, phosphatidylcholine, indole-3-acetaldehyde, dichloroethane, hydroxycinnamic acid, and indole-3-methanol. *Catenibacterium* showed a negative correlation with phosphatidylcholine and phenylalanine, whereas *Azoarcus* was positively correlated with phosphatidylcholine, indole-3-acetic acid, butanoic acid, and phosphatidylcholine. *Pyramidobacter* showed a negative correlation with phenylalanine. *Raoultella* was negatively correlated with monolinolein and phenylalanine. *Staphylococcus* showed a positive correlation with phosphatidylcholine and a negative correlation with cholanic acid. *Haemophilus* was positively correlated with benzaldehyde and erythrina alkaloids and negatively correlated with amygdalic acid, phenylalanine, and glycine. *Thiothrix* was positively correlated with dichloroethane and negatively with 3-dehydrocarnitine. Within the Actinobacteria, Gaiellales_unclassified was positively correlated with phosphatidylcholine, indole-3-acetaldehyde, pyridinecarboxylic acid, and myristoyl glycine while showing a negative correlation with benzaldehyde and erythrina alkaloids.

Porphyromonadaceae_unclassified within the Porphyromonadaceae family was positively correlated with genistein and calcium pentanoate hydrate and negatively correlated with omega-3 fatty acids. *Coprococcus* exhibited a positive correlation with phosphatidylcholine and monolinolein. Porphyromonadaceae was positively correlated with caffeic acid, genistin, and 3-methyl-2-oxovaleric acid and negatively correlated with docosatrienoic acid. *Eubacterium* showed significant positive correlations with lysophospholipid, cadaverine, 1-amino-1-cyclobutanecarboxylic acid, ginkgolic acid, and lysophosphatidylcholine.

## 4 Discussion

In this study, Salmonella Pullorum (SP) was detected using the rapid plate agglutination (RPA) test and PCR-based methods (fecal and anal swab samples). The RPA test, known for its specificity, confirmed the presence of antibodies against SP, while PCR offered a more sensitive approach for detecting SP DNA directly from samples (Sichuan Provincial Department of Agriculture and Rural Affairs, 2019). Combining these methods is vital for early PD detection and controlling its spread, especially in asymptomatic carriers, highlighting the importance of comprehensive surveillance strategies in poultry farms. In addition, This study investigated the effects of SP infection on the host's intestinal flora and metabolites using 16S rDNA sequencing and metabolomics analysis, revealing significant alterations in the structure and metabolic functions of the microbiota. These findings provide new perspectives for understanding PD pathogenesis and the potential of intestinal microecology in disease management.

Through histopathological analysis using H&E staining, varying degrees of inflammatory responses and hemorrhagic phenomena were observed in the cecum, spleen, liver, and lungs of Group P. Therefore, it was hypothesized that these changes may be related to the gut microbiota and metabolites. Alterations in the gut microbiota can influence pulmonary inflammation, immune responses, and disease susceptibility, potentially through immune modulation or metabolic byproducts (Saint-Martin et al., 2022). The gut microbiota and metabolites in the cecum play a significant role in modulating the intestinal immune function in broilers, which in turn can affect growth traits (Bortoluzzi et al., 2017). The observed overexpression of Bacteroidota and Verrucomicrobiota in Group P may be attributed to their roles in maintaining gut health and suppressing inflammation (Zafar and Saier, 2021; Zhang et al., 2021). Furthermore, previous studies indicated that metabolites such as LysoPG 19:1 and LysoPI 14:0 can influence fat deposition in broilers (Liu et al., 2022). In this study, these metabolites were associated with cecal inflammation. Therefore, it was speculated that LysoPG 19:1 and LysoPI 14:0 may primarily affect the inflammatory response in the cecum, subsequently impacting the digestive system and leading to fat deposition in broilers.

Our results showed that SP infection led to a significant decrease in the diversity of intestinal microbiota. Several studies showed that the diversity of the intestinal flora is an important marker of the host's health and that higher flora diversity usually implies a more stable and disease-resistant intestinal environment (Liu et al., 2015). Interactions between the gut microbiota and the host's immune system begin at birth, with evidence suggesting that the gut flora may influence the development of the immune system, whereas the immune system, in turn, affects the composition of the gut microbiota (Nicholson et al., 2012). Imbalances in the host's gut flora can activate immune pathways and play a key role in disease progression (Dheer et al., 2020). Modulation of the intestinal flora can improve poultry growth performance, reduce gut inflammation, and restore intestinal mucosa damage (Wang et al., 2019). The gut microbiota plays a key role in immune function, digestion, and metabolism. Altered or destabilized microbiota and changes in its biodiversity characterize many gastrointestinal and metabolic diseases (Aziz et al., 2013). Therefore, the decreased flora diversity caused by SP infection may weaken the host's immune defenses and make it easier for pathogenic bacteria to colonize. This finding suggests that enhancing or restoring flora diversity may be an effective strategy in treating and preventing PD. SP infection alters the colonization of the host's intestinal flora by disrupting beneficial bacterial populations, impairing the host's immune response, and facilitating pathogen colonization (Stecher et al., 2007; Thiennimitr et al., 2012; Lawley et al., 2008), also confirming the importance of this idea.

This study observed a significant increase in Bacteroidota and Verrucomicrobiota abundance in Group P, whereas Firmicutes and Actinobacteria were more abundant in Group N. The changes in these phyla may reflect the reconstruction of the intestinal microecology after SP infection. Firmicutes and Actinobacteria are generally considered beneficial because of their roles in the host's metabolism and immunomodulation. The higher abundance in Group N may be linked to a healthier status, whereas Bacteroidota and Verrucomicrobiota abundance the increased relative abundance of Verrucomicrobiota may be a sign of an imbalance in the intestinal microbial ecosystem, a change that may be associated with the pathologic course of SP infections. When pathogens have adapted to survive in the antimicrobial defense system that develops in the inflamed intestinal lumen, it would allow SP to take advantage of the inflammation to compete with the intestinal microbiota and spread through the oral-fecal route (Santos et al., 2009). Furthermore, the host's infection with SP results in changes in the gut flora and the abundance of 39 genera of bacteria (Ding et al., 2021), suggesting that SP may disrupt the normal microbial balance through a mechanism that encourages the proliferation of specific phyla.

At the genus level, SP infection resulted in significant changes in the relative abundance of multiple genera. There was an increase in *Akkermansia* abundance in Group P, which may be related to the disruption of the intestinal mucosal barrier. *Akkermansia* was more competitive in certain inflammatory environments (Bian et al., 2019; Belzer and de Vos, 2012), so its elevated abundance may respond to SP infection triggering intestinal inflammation. However, *Akkermansia* was also suggested to be beneficial to the host's health under certain conditions (Cani et al., 2022), suggesting that it may play a dual role in different infection states. Future studies could further explore the function of *Akkermansia* in the infected state and elucidate its different mechanisms of action in inflammation and recovery.

This study investigated the effects of SP infection on cecum metabolites in 12 white-feathered broilers from Groups P and N using non-target metabolome sequencing. Seventy-seven differential metabolites were identified at the secondary metabolite level. The microorganisms in the gut and the numerous metabolites produced by microbial-host interactions constitute the basic environment in the gut and play an important role in maintaining the host's health. Metabolomics has become an important component of systems biology capable of quantifying the subtle dynamics of an organism's metabolic pathways due to changes in pathophysiology, nutrition, and epigenetic status (He et al., 2011). In this study, some specific metabolites (e.g., 2,4-dimethylbenzaldehyde, 3a,6b,7b,12a-tetrahydroxy-5b-cholanoic acid, and LysoPG 19:1) were significantly overexpressed in the cecum of the infected group, suggesting that these metabolites may have a specific function in the infection process. For example, an increase in cholanoic acid may be associated with enhanced intestinal permeability and the activation of inflammatory responses (Ning et al., 2024; Calzadilla et al., 2022). Therefore, an increase in cholanoic acid expression triggered by SP infection may lead to increased intestinal inflammation or be a form of self-regulation by the host, and the exact reasons need to be further investigated.

The elevated expression of metabolites such as methyl dihydrojasmonate, lenticin, and deoxyadenosine monophosphate may indicate a redistribution of the host's metabolism, potentially serving as a self-regulatory mechanism for energy and resource allocation during infection (Wilk et al., 2024; Zhang et al., 2023). The increase in the expression of these metabolites suggests that the host may reorganize its metabolic network in response to pathogen-induced metabolic stress during infection. This metabolic reorganization may not only be a passive response to infection but also an active strategy adopted by the host to resist pathogens and maintain metabolic homeostasis.

This study revealed complex and fine-grained interrelationships between the gut microbiota and host metabolites. Through Spearman correlation analysis of 47 differential genera and 68 metabolites, multiple microflora genera were significantly associated with specific metabolites. The significant correlations not only suggested that gut microbes may play an important role in the host's metabolic function but also implied that specific genera may be involved in regulating metabolic health by influencing the host's metabolic pathways. Benefits of the joint analysis of metabolomics and other related histologies for diagnosing and treating diseases (Bjerrum and Nielsen, 2019). g__Corynebacterium was positively correlated with phosphocholine and indole-3-acetaldehyde but negatively correlated with tetrahydroxycholanic acid. This phenomenon may reflect the dual regulatory role of *Corynebacterium* in choline metabolism and indole derivative metabolism (Cho et al., 2024). A plausible explanation is that such genera may regulate choline metabolism while inhibiting the production of certain bile acids through their metabolic activities, with potential implications for the host's lipid metabolism and intestinal health. g__Roseobacter with indole-3-propionic acid, phosphatidylcholine, and indole-3-acetaldehyde were positively correlated, suggesting that it may play a central role in various metabolic pathways, especially for the metabolism of aromatic compounds and phospholipids. The activity of this genus may promote the production of specific aromatic metabolites in the intestinal microenvironment, which may be important for the host's neural activity and immunomodulation.

In summary, this study reveals the profound effects of SP infection on the host's gut flora and metabolites through combined microbiota and metabolomics analyses, providing new insights into understanding the relationship between the gut flora and the host's metabolism. The results suggest that specific genera of bacteria may be involved in regulating gut health through the choline and aromatic compound pathways. These findings not only deepen the understanding of PD pathogenesis but also provide theoretical support for microbiota-based disease prevention strategies in poultry.

## 5 Conclusion

SP infection leads to varying degrees of inflammatory cell infiltration in the spleen, liver, lungs, and cecum tissue of broilers. SP infection affects gut metabolites, with 11 metabolites, including 2,4-dimethylbenzaldehyde, showing significantly elevated expression levels in the gut after infection. SP infection also alters gut microbial diversity, significantly increasing the abundance of Verrucomicrobiota and Bacteroidota while reducing the abundance of Firmicutes, Actinobacteriota, Desulfobacterota, Proteobacteria, and Synergistota. Combined analysis revealed significant correlations between the gut microbiota and the host's metabolites, identifying associations between 28 bacterial genera and 38 metabolites. Specific genera, such as *Corynebacterium* and *Roseobacter*, are particularly influential on metabolite profiles.

## Data Availability

The original data of this study has been uploaded to the GSA database and can be accessed directly at https://ngdc.cncb.ac.cn/gsa/. Bioproject number: PRJCA033472.
